# Anthrax outbreak in a Swedish beef cattle herd - 1st case in 27 years: Case report

**DOI:** 10.1186/1751-0147-52-7

**Published:** 2010-02-01

**Authors:** Susanna Sternberg Lewerin, Marianne Elvander, Therese Westermark, Lisbeth Nisu Hartzell, Agneta Karlsson Norström, Sara Ehrs, Rickard Knutsson, Stina Englund, Ann-Christin Andersson, Malin Granberg, Stina Bäckman, Per Wikström, Karin Sandstedt

**Affiliations:** 1 Department of Disease control & Epidemiology, National Veterinary Institute, SE-751 89 Uppsala, Sweden; 2 Varberg Veterinary Practice, Engelbrektsgatan 20, SE-432 42 Varberg, Sweden; 3 Eurofins Food & Agro Sweden AB, Box 9024, SE-291 09 Kristianstad, Sweden; 4 Swedish Board of Agriculture, SE-551 82 Jönköping, Sweden; 5 Department of Bacteriology, National Veterinary Institute, SE-751 89 Uppsala, Sweden; 6 Department of Animal Health and Antimicrobial Strategies, National Veterinary Institute, SE-751 89 Uppsala, Sweden; 7 CBRN Defence and Security, Swedish Defence Research Agency, SE-901 82 Umeå, Sweden

## Abstract

After 27 years with no detected cases, an outbreak of anthrax occurred in a beef cattle herd in the south of Sweden. The outbreak was unusual as it occurred in winter, in animals not exposed to meat-and-bone meal, in a non-endemic country.

The affected herd consisted of 90 animals, including calves and young stock. The animals were kept in a barn on deep straw bedding and fed only roughage. Seven animals died during 10 days, with no typical previous clinical signs except fever. The carcasses were reportedly normal in appearance, particularly as regards rigor mortis, bleeding and coagulation of the blood. Subsequently, three more animals died and anthrax was suspected at necropsy and confirmed by culture and PCR on blood samples.

The isolated strain was susceptible to tetracycline, ciprofloxacin and ampicillin. Subtyping by MLVA showed the strain to cluster with isolates in the A lineage of *Bacillus anthracis*.

Environmental samples from the holding were all negative except for two soil samples taken from a spot where infected carcasses had been kept until they were picked up for transport.

The most likely source of the infection was concluded to be contaminated roughage, although this could not be substantiated by laboratory analysis. The suspected feed was mixed with soil and dust and originated from fields where flooding occurred the previous year, followed by a dry summer with a very low water level in the river allowing for the harvesting on soil usually not exposed. In the early 1900s, animal carcasses are said to have been dumped in this river during anthrax outbreaks and it is most likely that some anthrax spores could remain in the area.

The case indicates that untypical cases in non-endemic areas may be missed to a larger extent than previously thought. Field tests allowing a preliminary risk assessment of animal carcasses would be helpful for increased sensitivity of detection and prevention of further exposure to the causative agent.

## Background

Anthrax is a bacterial infection that affects both animals and humans. It is caused by the gram positive, rod-shaped spore-forming bacterium *Bacillus anthracis*. Fully virulent isolates contain two plasmids, pX01 and pX02. The former encodes the tripartite protein exotoxin complex, consisting of lethal factor, protective antigen and oedema factor, and the latter encodes the poly-D-glutamic acid capsule [1,2]. In an environment with elevated CO_2 _levels, as in an infected animal, the virulence factors are induced and sporulation is inhibited [1]. When the bacteria are released outside the infected host, as when blood oozes from a carcass, the lower CO_2 _levels in open air induce sporulation, which allows the organism to survive in the environment for long periods of time [1]. The spores are extraordinarily resistant to extremes of pH, heat and cold, desiccation and various chemical agents [3,4]. The period of survival of anthrax spores in the environment can be very long [5,6], reportedly up to 200 years [7], and is affected by pH, water activity, temperature and the presence of nutrients.

Due to the long persistence of anthrax spores in soil, no country can claim absolute freedom from the agent, but regular outbreaks usually occur in limited geographic regions. Endemic foci exist in most parts of the world, including Africa, Asia, United States and Australia [8,9] and regular vaccination is practised in many of these areas.

The susceptibility to infection varies among host species, with cattle and sheep being the most susceptible, followed by goats and horses, humans are regarded as intermediate in susceptibility and swine and carnivores relatively resistant [1].

Spores from the environment enter the host via ingestion or inhalation, are taken up by macrophages and transported to lymph nodes where the spores germinate into vegetative bacteria that multiply quickly and escape into the bloodstream, causing systemic reactions due to the release of toxin [1]. Cutaneous infection also occurs (this is the most common form in humans) and may give rise to a local oedema that develops into a necrotic lesion and/or progress to a systemic infection [4]. The acute form of the disease, the most common in cattle and sheep, is seen only as sudden death, where the carcass is typically characterised by dark non-coagulated blood oozing from orifices, lack of rigor mortis and quick decomposition [1]. Prior symptoms, if observed, may include fever, listlessness, oedema and bleeding from mucous membranes [4]. The signs observed in subclinical cases vary but may include oedema of the throat and neck and/or gastrointestinal symptoms. In some less susceptible host species, gastrointestinal infection may occur without systemic involvement and symptoms caused by toxins released in the intestinal canal, by bacteria that multiply in the intestines [1]. *B. anthracis *is susceptible to several antimicrobials, but therapy has to be administered early in the course of infection, since the toxin effects are not influenced by antimicrobials and symptoms caused by already released toxin will persist in spite of therapy.

The incubation period varies by host species, route of infection and other factors but is estimated to 1-14 days in natural infection of cattle [4]. The infectious dose also depends on host species and route of infection and estimates vary [4]. Cattle may be difficult to infect by the parenteral route while readily infected when given anthrax spores in feed [10]. The number of spores required for oral infection of cattle has not been reliably determined and the assessment of risks from environmental exposure is therefore difficult.

Now that meat and bone meal is no longer fed to ruminants and swine, this formerly common route of infection has been practically eliminated. The most common cause of infection these days is exposure of grazing animals to environmental spores persisting in soil, but rare outbreaks in cattle housed in barns have been reported [4]. The successful prevention of anthrax in many parts of the world has led to the disease almost being forgotten by both farmers and veterinarians, a fact that may lead to failures in clinical surveillance and thus underreporting of occurrence [4].

The occurrence of anthrax is closely linked to climate [4,8]. Changes in climate with warmer temperature and more incidents of extreme weather that interfere with soil surface may cause more frequent exposure of ruminants to old anthrax spores and thus new outbreaks in areas currently regarded as "free". The risk of re-occurrence of anthrax is hard to assess, due to lack of detailed information about where infected carcasses have been buried and lack of data on infectious doses required for inhalation and ingestion by grazing animal species. In spite of the long-standing knowledge of the disease some crucial data on pathogenesis is still missing and a lot of what is known relies on theory rather than scientific data [4].

As in most European countries, anthrax was common in Swedish livestock in the first half of the 20th century. A large outbreak, associated with imported meat-and-bone meal, occurred in the county of Halland in the 1950s [11]. However, in the latter part of the 20^th ^century the disease was regarded as practically extinct. In most areas in Sweden, the soils are not very alkaline [12] and the general conception has been that soil contamination may not be a major risk in this country. However, the level of environmental contamination is also likely to depend on the management of previous anthrax cases. Anthrax is included in the Swedish Epizootic Act [13], which means that any suspicion is notifiable and that the veterinary authorities are obliged to undertake control and eradication measures in case the infection is detected. The absence of detected cases for several decades has strengthened the perception that eradication measures along with favourable environmental conditions may have succeeded in reducing soil contamination to a negligible level. A search for old data has revealed a very high number of anthrax cases in several parts of the country not so long ago and most carcasses appear to have been buried. Thus, the perceived risk from soil may have been underestimated.

In 1981, a single case occurred in a dairy farm in the county of Uppland, most likely associated with exposure to spores from a soil heap that had been moved just before the onset of symptoms in the cow.

Twenty-seven years later, an outbreak occurred in a beef herd in the county of Halland, in the South of Sweden. The outbreak was unusual as it occurred in winter, in animals housed on deep straw and fed only roughage, in a non-endemic country.

## Case Presentation

### The herd

The affected herd consisted of 45 beef cows of mixed breed and their offspring, including calves and young stock. In total there were about 90 animals on the holding. The calving period was mainly in autumn. The animals were kept on pasture during the warmer part of the year and in a barn on deep straw bedding during winter. No supplementary feeding was given on pasture and during winter the animals were fed only roughage in the form of poor quality silage. No minerals were fed. During winter, the animals had access to a small paddock just outside the barn during daytime.

### Clinical history

The animals were brought indoors in mid-October in 2008. They were vaccinated against bluetongue serotype 8 with an inactivated vaccine within the official Swedish vaccination campaign [14] on the 25^th ^of November. On the 29^th ^of November one animal (animal 1) died without any observed previous symptoms. The owner of the herd called his veterinarian to ask whether it could be a side effect of the vaccination but as this was rejected, he sent the carcass for routine destruction. The carcass was reported by the owner to be normal in appearance, with ordinary rigor mortis, no abnormal bleeding or abnormal appearance of any blood that was observed, and the carcass collector had the same recollection.

On the 4^th ^of December another animal (animal 2) died and two more (animals 3 and 4) were listless and feverish and the owner called his veterinarian. The animals were found to have fever, a high pulse and increased, rattling, breathing sounds and were treated with danofloxacin and meloxicam. The carcass was sent for necropsy. It was a Thursday, and the veterinarian made sure that the transport would deliver the carcass to the regional laboratory the next day and that it would be necropsied immediately so as to ensure a good quality of the investigation. However, the carcass did not arrive to the regional laboratory until the following Monday (8^th ^of December). On Thursday evening (the 4^th ^of December) another animal (animal 5) died and the owner cut it open and brought the liver, spleen, lungs and heart into the local veterinary clinic for examination. During the night, the two animals that had been treated the previous day (animals 3 and 4) also died. On the 5^th ^of December, the owner contacted his veterinarian who at this time also learned that the carcass sent for necropsy (animal 2) had not arrived in the laboratory. She then contacted the National Veterinary Institute (SVA) for advice. During this consultation, anthrax was discussed as a possible diagnosis but was regarded as less likely due to the feeding history and the lack of typical signs (as reported by the owner, the carcass collectors and observed by the veterinarian herself) in the carcasses. Other possible causes that were discussed were pasteurellosis, clostridiosis, poisoning and mineral depletion. It was decided to take samples for histology and microbiology from the next animal that died if it could not be sent directly for necropsy. In the evening a calf died (animal 6), but the owner did not report it at the time and only sent it for destruction.

On the 7^th ^of December the owner culled one animal (animal 7) that was, he thought, on the verge of dying, and took out samples of spleen, lung and liver and sent them to SVA for culture and histology. However, the receiving laboratory did not realise that the animal had been culled and not died by itself and thus assumed that a diagnosis of septicaemia would have been readily made by bacterial culture.

On the 8^th ^of December the missing carcass (animal 2) arrived in the regional laboratory. Due to decomposition of the carcass a full necropsy could not be performed but a swab sample was taken from the spleen and sent to SVA for culture.

On the night between the 9^th ^and the 10^th ^of December another animal died (animal 8) and a separate transport was arranged to take the carcass directly to the regional laboratory for necropsy. When the vehicle arrived to the farm two more animals (animals 9 and 10) had died and were also taken to the laboratory. When the carcasses arrived in the laboratory and the first one was opened, the appearance (massive internal bleeding and non-coagulated blood) made the investigating veterinarians suspect anthrax and take actions accordingly. SVA was contacted and the other two carcasses were left unopened. It was decided to send blood samples from all three animals by courier to SVA and the samples arrived on the following morning (11^th ^of December).

After the diagnosis of anthrax was confirmed on the 12^th ^of December, environmental samples were taken on the farm. These included various dust samples from stored roughage and straw for bedding and from feeding troughs as well as soil samples from areas just outside the barn where infected carcasses had been left on the ground until they were picked up for transport. The dust samples were collected both by hand (10-20 samples from various storage areas) and with a small vacuum cleaner (some 20 samples from packed roughage and feeding troughs). Soil samples were collected manually (5 samples from 2 spots).

All people potentially exposed to bacteria and/or spores were given postprophylactic treatment with antibiotics. The remaining animals were treated with long-acting antibiotics, to reduce the risk of further transmission, and subsequently culled. This was due to the practical difficulties in keeping them on the farm or transporting them elsewhere during the cleanup work on the holding. The animal holding, the laboratory performing the necropsies and the rendering plant that had received the carcasses from animals 1-7, and also received the remaining culled animals, were thoroughly cleaned and disinfected. The carcasses from animals 8-10, plus two more animals (animals 11 and 12) that died on the farm after the diagnosis had been confirmed, were incinerated at SVA.

### Laboratory investigations

All laboratory investigations except for the soil analyses and MLVA typing were performed at SVA. All samples analysed before the suspicion of anthrax arose were handled by routine procedures. Necropsy and histology were performed according to standard procedures. Routine culture was made on blood agar plates incubated at 37°C for 24 h in aerobic conditions.

The blood samples from the three suspect cases (animals 8, 9 and 10) were investigated by microscopy, culture, and PCR.

The specimens were not entirely fresh, since the blood samples arrived to the laboratory > 24 h after the death of the animals.

Smears of blood were dried, fixed and stained with polychrome methylene blue. Methylene blue solution was prepared as follows: 0.5 g of methylene blue was dissolved in 25 g of 96% ethanol; 0.01% NaOH was mixed with the methylene blue solution to a final volume of 100 ml. This was left to stand exposed to the air, with occasional shaking, for at least 1 year to oxidize and mature ("old methylene blue"). Smears were examined with respect to bacterial morphology and presence of capsule.

In order to demonstrate growth of *B. anthracis *the samples were also spread on agar (Oxoid, Cambridge, UK) supplemented with 5% horse blood as well as agar with 1.6% bromcresolepurpur (Merck, Darmstadt, Germany) and 20% lactose (Merck, Darmstadt, Germany) and incubated aerobically in 37°C overnight.

#### PCR

PCR on the spleen swab, blood and dust was performed at SVA. DNeasy Blood and Tissue kit (Qiagen, Hilden, Germany) was used for DNA extraction with a slight modification of the manufacturer's protocol for isolation of DNA from gram-positive bacteria. Dust samples were cultured before extraction. Approximately 2 g of sample was added to 18 ml Luria-Bertani (LB) broth and heated at 70°C for 30 min. Dilution of the sample was done by transferring 1 ml to 10 ml LB broth. Both broths were incubated at 37°C over night. From the diluted sample 1 ml was centrifuged at 6000 × g for 2 min. The pellet was resuspended in 180 μl from the undiluted culture and the suspension was extracted as described above. Three real-time PCR assays were used to detect *B. anthracis *DNA. The SYBR Green based assays target three genes; i.e. the *rpoB *gene on the chromosome and the virulence genes *lef *and *cap *located on the pXO1 and the pXO2 plasmids. Primers targeting *cap *(primers iQBa2F; 5'-CTTAAATCACTTTTGCTTGCTTTTTG and iQBa2R; 5'-TGCAGCTGAGCCATTAATCG), *lef *(iQBa3F; 5'-AAGAAGGATATGAACCCGTACTTGTAA and iQBa3R; 5'-AAACGTTCAGTGCCTTTTCAGTATT) and *rpoB *(iQBa4F; 5'-GAAGGACGATACAGACATTTATTGG and iQBa4R; 5'-ACCGCAAGTTGAATAGCAAG) for *B. anthracis *were used. PCR reactions were performed in a final volume of 25 μl containing 5 μl DNA template, 2 × PowerSYBR^® ^Green PCR master mix (Applied Biosystems, Foster City, USA), forward primer 0.4 μM and reverse primer 0.4 μM and 0.2 mg/ml BSA (Sigma, Saint Louis, USA). Temperature cycling conditions were as follows: 10 min denaturation at 95°C; 40 cycles of 95°C for 15 s, 60°C for 60 s and melting curve 95-60°C.

#### Genetic analyses of nucleic acid extracted from soil

Nucleic acid was extracted from five soil samples. Three of them were taken from a spot where carcasses of a cow and a calf (animals 11 and 12) had been left lying, and the remaining two soil samples were taken in a paddock next to the buildings where the animals were housed. SoilMaster DNA Extraction Kit (Epicentre Biotechnologies, Madison, Wisconsin, USA) was used for DNA extraction, following the manufacturer's protocol.

PCR analyses were done in triplicates from the extracted nucleic acid material. Primers targeting *cap *(primers iQBa2F and iQBa2R) and *lef *(primers iQBa3F and iQBa3R) were used. All soil samples were spiked with rat-DNA as a positive internal control of the DNA extraction efficiency and detected using the primers; iQFPrat36B4; 5'-GCCCAGAGGTGCTGGACAT and iQRPrat36B4; 5'-ATTGCGGACACCCTCTAGGA. PCR reactions were performed as follows; total DNA from soil was amplified in a final volume of 20 μl containing 2 × Fast Cycling SYBR^® ^Green qPCR reaction mix (Quanta Biosciences, Gaithersburg, Maryland, USA), forward primer 0,4 μM and reverse primer 0,4 μM. Temperature cycling conditions were as follows: 10 min denaturation at 95°C; 40 cycles of 95°C for 15 s, 60°C for 60 s and melting curve 95-60°C.

#### MLVA (Multi Locus Variable tandem repeats Analysis) typing of the three animal strains

*B. anthracis *DNA from three isolates from animals 8, 9 and 10, respectively, were prepared as described above for blood. A MLVA using 16 markers, viz. vrrA, vrrB1, vrrB2, vrrC1, vrrC2, CG3, BAMS1, BAMS3, BAMS5, BAMS13, BAMS21, BAMS25, BAMS34, BAMS44, BAMS51, and BAMS53, previously reported [15-17], was done on the genetic material from all three animal isolates with some modifications compared to Lista [17]. Briefly, singleplex PCR reactions were performed as follows; 10 ng DNA were amplified in a final volume of 25 μl containing 1xBuffer for DyNAzyme DNA polymerase (Finnzymes, Espoo, Finland), dNTP 0,15 mM (Finnzymes, Espoo, Finland), DyNAzyme DNA polymerase II 0,6 U (Finnzymes, Espoo, Finland), forward primer 0,4 μM and reverse primer 0.4 μM. The thermal cycling conditions were initial step, 96°C, 3 min for polymerase activation; PCR (40 cycles), 95°C, 20 s for denaturation, 60°C, 30 s for annealing and 65°C, 2 min for extension. The reactions were terminated by a final incubation at 65°C for 5 min.

After diluting the PCR products 1/5, 1 μl was added to 40 μl of Sample Loading Solution (Beckman-Coulter, Fullerton, California, USA) containing 0.32 μl MapMarker 1000 (Bioventures, Inc., Murfreesboro, Tennessee, USA). The samples were separated on a CEQ 8800 automatic DNA Analysis System (Beckman-Coulter, Fullerton, California, USA) with the following conditions: denaturation 90°C for 120 s, inject 2.0 kV for 30 s, separation 6.0 kV for 60 min.

The MLVA profiles were compared, using a web-based tool, to a large global MLVA database (http://minisatellites.u-psud.fr/MLVAnet/) containing typing data from *B. anthracis *strains.

#### Antimicrobial susceptibility testing

Susceptibility to antimicrobials was tested following the standards for microdilution of the Clinical and Laboratory Standards Institute [18,19]. Minimum inhibitory concentration (MIC) was recorded as the lowest concentration of the antimicrobial that inhibits bacterial growth. The antimicrobials tested were: ampicillin (representative for penicillin), ciprofloxacin, gentamicin, streptomycin and tetracycline, based on the EMEA/CPMP guidelines [20].

### Results of investigations

#### Macroscopic examination (in the local veterinary clinic) of the organs of animal 5

No specific findings were observed, apart from bleeding in an area of the inner wall of the left chamber and atrium of the heart, some bleeding in the lungs and fat deposition in one liver lobule. The blood appeared normal in colour and coagulation.

#### Histology and culture (at SVA) on organs from animal 7

No specific histological lesions were seen, only haemorrhages in examined organs. Routine culture from the lung revealed no bacterial growth.

#### Bacteriological examinations (at SVA) of spleen swab from animal 2

Routine culture from the swab revealed a mixed flora with no specific growth. PCR on the swab, performed later, was positive for pXO1, pXO2 and the chromosomal markers. The C_t _values were in the range of 21-23 for all three targets.

#### Necropsy findings (in the regional laboratory) in animal 8

The carcass appeared normal before opening, with no extensive bleeding form orifices. The necropsy revealed massive internal bleeding with non-coagulated blood in almost every organ. Typical signs were seen such as petechia in mucuous membranes, connective tissue oedema and a large and friable spleen with a dark cut surface reminiscent of blackberry jam. Severe subserosal bleedings were noticed on the diaphragm, as well as subpleural bleedings on the lung surface. The blood was non-coagulated and dark. The content of the jejunum was watery and blood-stained. On the ventral side of the neck there was a large haemorrhagic oedema.

#### Bacteriological examination (at SVA) of blood from animals 8, 9 and 10

Direct smears of blood showed numerous bacillus-shaped rods and sparse occurrence of other bacteria. However, the presence of capsule could not be demonstrated. Cultures from all three animals showed heavy growth of *B. anthracis *mixed with contaminating flora. The colonies were typical for *B. anthracis*; grey, non-haemolytic, with a ground-glass moist surface. Microscopy revealed spore forming rods, and a capsule could be visualised after culture for 5 h in horse serum and staining with polychrome methylene blue. The real-time PCR assay was positive for *B. anthracis *since all three genes were detected. The C_t _values from animal 8, 9 and 10 were in the range of 11-18 for all three targets. The C_t _values indicated a high concentration of *B. anthracis *cells in the blood. DNA was sent to the Centre for Microbiological Preparedness at SMI for a second real-time PCR confirmation and the result showed positive results for *B. anthracis *DNA.

According to the MIC interpretive standard from CLSI for potential agents of bioterrorism the isolates were found to be susceptible to ciprofloxacin and tetracycline with MIC-values of 0.12 μg/ml and 0.25 μg/ml, respectively [19]. The MIC-value for ampicillin was 0.25 μg/ml, indicating that the anthrax strain was susceptible using the MIC interpretive standard for penicillin. The MIC-value for gentamicin was 0.25 μg/ml and for streptomycin 2 μg/ml. For these two antimicrobials there is no data available for interpretation of susceptibility.

#### Bacteriological examination of environmental samples

None of the dust samples were positive. Two out of three soil samples taken practically on the same spot (where animals 11 and 12 had been lying) were positive for both the *cap *and the *lef *gene, while the third sample was negative. Two other soil samples taken on another spot were both negative.

#### Subtyping

The three animal isolates showed the same MLVA profile. No perfect match to other published profiles was found. However, this study's profile clustered with isolates in the A lineage that, unlike other major lineages, is known be present throughout the world [21].

## Discussion and Conclusions

This case illustrates the difficulties in detecting a disease that has been absent for a long period of time. The absence of typical signs such as dark blood failing to clot, or lack of rigor mortis, in combination with a non-typical history of animals kept indoors fed only roughage, caused a delay in the diagnosis that led to a number of potential human exposures and consequent antibiotic treatments. Most cases reported from other countries are in grazing animals and "barn anthrax" is rarely reported now when meat-and-bone meal is no longer fed to ruminants [4]. However, one similar case has been described [4] where heifers indoors on a strict hay diet contracted anthrax via contamination of the hay. In that case, spores could be detected in the hay. In the current case, no samples of dust from either hay or straw were positive, in spite of great efforts to obtain representative samples. The culture method that was used before PCR on these samples had not been evaluated earlier and the detection limit of the method is unknown. It is most likely that low concentrations of contamination would not have been detected. Any such contamination is believed to have been of a low concentration possibly originating from fields close to the river Viskan. In these fields, flooding occurred the previous year and the next year there was a draught with a very low water level in the river allowing for the harvesting on soil not usually exposed. According to the farmer, the feed in question was mixed with soil and dust and this was also obvious at the time of sampling. In the early 1900s, animal carcasses are said to have been dumped in this river during anthrax outbreaks and it is most likely that some anthrax spores could remain in the area. The history of flooding followed by drought is typical for areas where old anthrax spores surface and cause outbreaks [4,22].

A low initial dose may be one reason for the less typical appearance of the first carcasses. Bleeding from orifices [22] or failure of the blood to clot is reported to be the most reliable sign of anthrax carcasses [23], but this was not seen in the field and was only obvious after opening the carcass of animal 8.

The lack of a laboratory diagnosis in animals investigated before anthrax was confirmed is, with hindsight, not surprising. Animal 2 had been transported for 4 days and was badly decomposed when the spleen swab was taken and thus the only remaining viable bacteria, that appeared on culture, were from the post mortem contamination flora. Later, when the swab was re-analysed by specific PCR, it was positive, demonstrating the need for this method when samples are from older carcasses. Animal 7 was culled by the owner and did not die from terminal bacteraemia. This was, however, not known by SVA at the time of investigation and thus the lack of bacterial growth on culture was at the time taken as contradicting the suspicion of anthrax. The histological findings were unclear and only indicated some type of infectious origin.

In contrast, the necropsy of animal 8 revealed typical signs and immediate direct smears performed in the regional laboratory had a more typical appearance than when smears were performed on blood sent to SVA. The absence of encapsulated *B. anthracis *in the latter smears could be due to the fact that the blood samples were not fresh, and the capsules present in the blood of diseased animals became subsequently decomposed.

Direct smears stained with "old methylene blue" have been widely used in the field and provide a quick preliminary diagnosis provided the carcass is fresh, a good microscope is available and the person performing the microscopic examination has some experience. It would not be practical in Swedish field circumstances today and even the regional laboratories rarely have adequate experience in microscopic examinations. However, rapid detection is important and a robust field test to replace direct smears would be of great benefit.

Both PCR and culture were used for the diagnosis and both methods are needed if a quick diagnosis on any sample, regardless of state of decomposition, is to be made while securing material for subtyping and antibiograms.

The positive PCR results from DNA extracted from the soil samples showed that it was possible to detect genetic material from *B. anthracis *in a well-known complex environmental matrix such as soil [24]. Our results demonstrate the potential of using PCR as a tool for mapping *B. anthracis*-contaminated areas and possibly elucidate the coordinates of the source. A careful sampling strategy is a prerequisite for such a study, and was beyond the scope of this reported work.

The MLVA results were not surprising. As the historical anthrax cases in Sweden were mainly associated with the feeding of imported meat-and-bone meal, it is to be expected that the spores remaining in Swedish soil are of the A lineage. Detailed subtyping of old anthrax strains has been performed in some parts of the world [25-27], revealing different pictures of genetic linkage between strains as well as possible clues to the origin of some strains. Unfortunately, the old anthrax strains that were formerly stored at SVA have been destroyed so no further studies can be made on old historical material unless old strains are recovered from the environment. Lacking detailed information on the exact location of old cattle graves, this is currently an unlikely scenario but efforts will be made to produce more detailed maps of the possible location of old anthrax spores.

In conclusion, the case described here may indicate that untypical cases in non-endemic areas are missed to a larger extent than previously thought. It may be argued that if these cases do not cause secondary cases there is no harm done. However, there is a need to detect any environmental contamination with anthrax spores so as to prevent future outbreaks. Further insight into the infective dose for grazing animals as well as the symptoms in such animals infected with low doses is needed to better predict risks from old spores remaining in non-endemic countries where the situation may change in the wake of climate change. Furthermore, a commercially available field test would be of great benefit for a preliminary risk assessment of animal carcasses, in order to prevent further exposure.

## Consent

As anthrax is included in the Epizootic Act (SFS 1999:657), the details of the case may be made publicly available and the Swedish veterinary authorities have the right as well as an obligation to report on all cases.

## Competing interests

The authors declare that they have no competing interests.

## Authors' contributions

SSL and ME took environmental samples in the herd, outlined the eradication efforts and gave advice on all aspects of the case as it evolved, TW was the field veterinarian in charge of the herd, LNH performed the necropsies, AKN handled all legal actions in the case, SEhrs adapted the DNA extraction for direct use on blood samples and perfomed the PCR on blood and dust samples in collaboration with RK, SEng performed the antimicrobial susceptibility testing, KS performed the bacteriological investigations, ACA and SB performed the DNA extraction and real time-PCR analysis from the soil samples, MG performed the MLVA analysis, PW did database comparisons and compiled the results from soil samples and MLVA.

Susanna Sternberg Lewerin wrote the manuscript and all other authors contributed with their respective parts of the text.
